# Mitral Valve Subacute Endocarditis Caused by Abiotrophia Defectiva: A Case Report

**DOI:** 10.3390/clinpract11010022

**Published:** 2021-03-02

**Authors:** Catarina Faria, Renato Guerreiro, Sofia Cruz, Marco Fernandes

**Affiliations:** 1 Internal Medicine, Hospital São Francisco Xavier—Centro Hospitalar Lisboa Ocidental, 1449-005 Lisbon, Portugal; renmjd.guerreiro@gmail.com (R.G.); marcofernandes21@hotmail.com (M.F.); 2 Internal Medicine, Hospital Vila Franca de Xira, 2600-009 Vila Franca de Xira, Portugal; cruz.sofiag@gmail.com

**Keywords:** subacute endocarditis, *Abiotrophia defectiva*, septic emboli

## Abstract

*Abiotrophia defectiva* is a rare agent of endocarditis and subacute presentation may delay the diagnosis. We present the case of a 41-year-old male who was admitted to the hospital for further investigation regarding a consumptive syndrome with microcytic anaemia. Past medical history included new-onset mitral insufficiency followed by an ischaemic stroke due to small vessel disease. Thoraco-abdominal computed tomography revealed a splenic infarction. In the presence of two ischaemic events associated with mitral valve disease of unknown aetiology, we considered the possibility of subacute endocarditis. Blood cultures were positive for *Abiotrophia defectiva*, and transoesophageal echocardiography confirmed the diagnosis. As a subacute presentation of endocarditis, the paucity of symptoms caused a five-month delay in diagnosis. New-onset valvular disease and a stroke in an otherwise healthy young patient should always prompt proper investigation. This case highlights several complications caused by septic emboli of undiagnosed and untreated endocarditis.

## 1. Introduction

*Abiotrophia defectiva*, former nutritionally variant streptococci, is part of the normal human flora and is present in the oral cavity and in the genitourinary and intestinal mucosae [1]. Nutritionally variant streptococci were first described in 1961 in the setting of endocarditis (Frenkel and Hirsch). It is thought to be responsible for 4.3–6% of all endocarditis, but estimates of their frequency are challenged by changes in nomenclature and difficulties in obtaining positive microbiology cultures [2,3].

Despite its insidious growth, it is a virulent bacterium [4] that is difficult to treat, with a failure rate of up to 40% [5]. Unlike other endocarditis agents, it is known for valve destruction, leading to heart failure and higher embolization rates. Its inherent resistance to routinely used antibiotics also contributes to increased mortality and morbidity, making timely diagnosis and prompt treatment essential for positive patient outcomes [4].

We present the case of *A. defectiva* subacute endocarditis diagnosed after two embolic events and the installation of severe mitral valve regurgitation.

## 2. Case Presentation

A 41-year-old male presented to a haematology appointment, after being referred by his general practitioner (GP), to investigate consumptive syndrome with microcytic anaemia.

He was previously healthy until October 2017, when a systolic murmur was diagnosed in a routine evaluation. Transthoracic echocardiography showed moderate to severe mitral insufficiency. At that time he was asymptomatic, but blood analysis showed a slight leukocytosis of 12,300/μL and a positive C-reactive protein (CRP) of 4.8 mg/dL.

**Figure 1:**
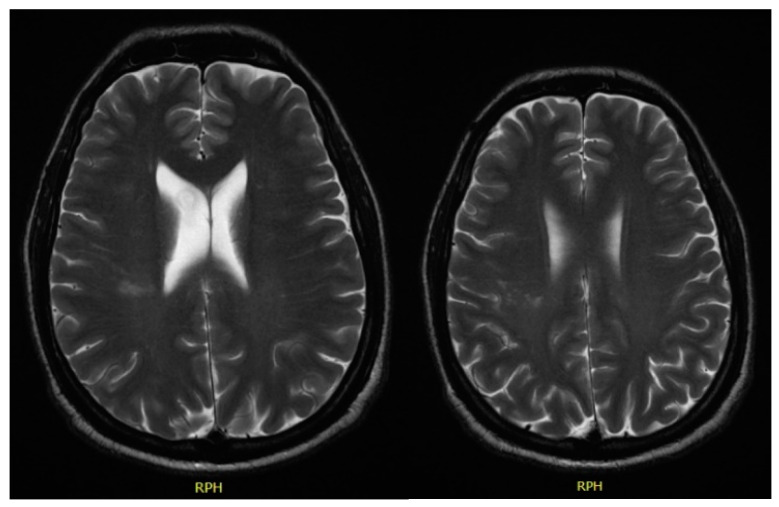
Cranioencephalic magnetic resonance with right ischaemic lacunar lesion in the corona radiata.

In December 2017, he was admitted to the emergency room with left central facial palsy and dysarthria. Cranioencephalic magnetic resonance showed an ischaemic lacunar lesion in the right *corona radiate* (Figure 1). Transoesophageal echocardiography (TEE) showed a prolapsed mitral valve, with suspected rupture of the anterior leaflet and severe regurgitation. Holter and ecodoppler of neck vessels were unremarkable. Blood analysis showed microcytic anaemia (haemoglobin 11.3 g/dL), with maintained leukocytosis of 11,000/μL and CRP of 10.2 mg/dL. A small vessel ischaemic disease was diagnosed. He was discharged and sent to cardiology and neurology appointments.

In February 2018, he went to a GP appointment complaining of medium effort dyspnoea, associated with palpitations. He denied having chest pain, orthopnoea, nocturnal paroxysmal dyspnoea or peripheral oedema. He also registered a weight loss of seven kilograms without anorexia and night sweats, without recording temperature. New blood analyses revealed aggravated microcytic anaemia with haemoglobin of 8.6 g/dL, leukocytosis of 13,700/μL, CRP of 9.1 mg/dL, and erythrocyte sedimentation rate of 63 mm/h. He had no visible gastrointestinal blood loss and denied abdominal pain or altered bowel movements. Upper endoscopy and colonoscopy were normal.

He was observed by a haematologist who admitted the patient to the internal medicine department for further investigation.

**Figure 2:**
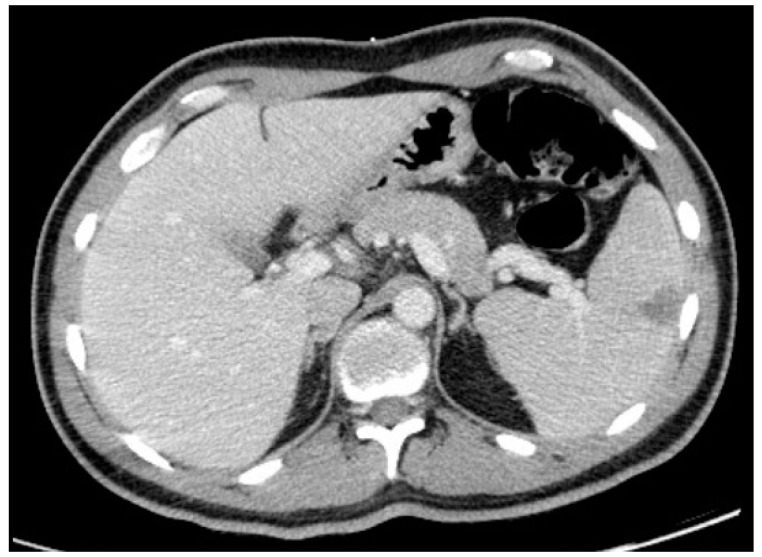
Thoraco-abdominopelvic CT showing splenic infarction.

Thoraco-abdominopelvic CT showed mediastinal lymphadenopathies, the largest being 6 mm, and no abnormalities in the lungs. It also revealed a triangular hypodensity in the spleen with 4.1 × 4 cm, suggesting splenic infarction (Figure 2).

In the context of two ischaemic events associated with mitral valve disease of unknown origin, we considered the possibility of subacute endocarditis. Blood samples were drawn and subjected to prolonged incubation culture analysis in search of HACEK bacteria. After a few days, the blood cultures were found to be positive for *Abiotrophia defectiva*. New TEE confirmed the diagnosis showing mitral valve thickening with large vegetation and severe regurgitation, with no complications observed, no abnormalities on the other valves, and preserved left ventricular ejection fraction.

**Figure 3:**
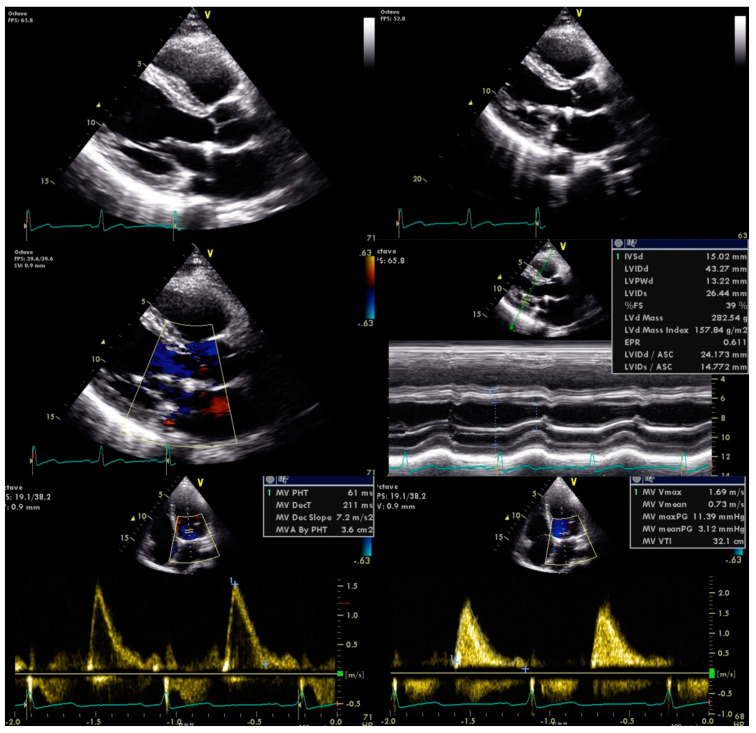
Transthoracic echocardiography post mitral valve plasty.

Antimicrobial therapy was promptly initiated with vancomycin and gentamicin for six weeks. He was also referred to the cardiothoracic surgery department for mitral valve plasty, with no further complications observed (Figure 3).

## 3. Discussion

This subacute presentation of endocarditis with an unusual microbiologic agent caused at least a five-month delay in diagnosis.

New-onset valvular disease in an otherwise healthy young patient, as well as an ischaemic stroke without cardiovascular risk factors, should always prompt proper investigation. The presence of leukocytosis and a positive CRP, alongside the first signs of illness, might have been an indication of a disguised infectious/inflammatory state.

Unlike infections caused by other streptococci, infective endocarditis by *A. defectiva* produces small vegetation [4], which might explain the missed diagnosis in the first TEE, when no vegetation was discovered. Unfortunately, no blood cultures were obtained at the time.

The appearance of a consumptive syndrome, always of great concern especially in a young patient, motivated further investigation. The finding of a splenic infarct in the thoraco-abdominopelvic CT, without any other significant findings, was the final clue connecting all the signs and symptoms and leading to the final diagnosis.

High clinical suspicion of subacute infectious endocarditis motivated a further search for rarer microorganisms in blood cultures allowing the growth of *A. defectiva*.

*A. defectiva* endocarditis is known for its complications, such as embolism, referred as up to 30% in some case series [6]. Our patient was mainly asymptomatic until the first major embolic complication, manifesting as a stroke. As discussed above, the absence of clinical suspicion for infective endocarditis and a TEE with no vegetation described delayed diagnosis.

The therapeutic regimen was discussed with the cardiothoracic team and was in line with the European Heart Association current recommendations. Treatment with antibiotics alone is frequently ineffective for *A. defective* endocarditis. Progressive valve destruction because of uncontrolled infection may lead to heart failure [7,8]. Despite the fact that our patient suffered from dyspnoea, we attributed this to the anaemia because he never had any other symptoms suggestive of heart failure.

Even though penicillin regimens are a therapeutic option, poor therapeutic response is not uncommon, accounting for up to 70% of some case series [9]. Given this, vancomycin and gentamicin for six weeks was the preferred treatment.

High antibiotic failure rates as well as persistent vegetation after two embolic events and severe mitral valve regurgitation led to valve surgery replacement, with no complications described.

The authors would like to point out the results of the early surgery versus conventional treatment for infective endocarditis (EASE) trial, which demonstrated that patients with severe valve disease and large vegetation had a more favourable outcome with an early-surgery approach [10].

## 4. Conclusions

*A. defectiva* is a rare but important cause of infective endocarditis. It can rapidly lead to valve destruction, causing heart failure, and can also result in distal organ embolism [4].

This case highlights several complications caused by the septic emboli of undiagnosed and untreated endocarditis.

Valve surgery replacement and adequate antibiotic coverage allowed full recovery, with no more complications described.
